# Identifying psychiatric comorbidities that occur following the introduction of hormonal contraception: a scoping review

**DOI:** 10.3389/fpsyt.2026.1783906

**Published:** 2026-06-01

**Authors:** Amélie Poirier, Juliette Fortier, Marie-France Marin, Marie Désilets, Alexandre Hudon

**Affiliations:** 1 Department of Medicine, Faculty of Medicine, Université de Montréal, Montréal, QC, Canada; 2 Department of Psychiatry and Addictology, Faculty of Medicine, Université de Montréal, Montréal, QC, Canada; 3 Centre de recherche de l’Institut universitaire en santé mentale de Montréal, Montréal, QC, Canada; 4 Department of Psychology, Université du Québec à Montréal, Montréal, QC, Canada; 5 Department of Psychiatry, Institut universitaire en santé mentale de Montréal, Montréal, QC, Canada; 6 Department of Psychiatry, Institut national de psychiatrie légale Philippe-Pinel, Montréal, QC, Canada

**Keywords:** anxiety, depression, hormonal contraception, mood disorders, oral contraceptive pills, progestin-only contraception, suicidal behavior, women’s mental health

## Abstract

**Introduction:**

Hormonal contraceptives are widely used by women of reproductive age, yet concerns persist regarding their potential effects on mental health. Although mood-related side effects are frequently reported, their prevalence, clinical significance, and variation across formulations remain unclear. This scoping review examined associations between hormonal contraception and psychiatric outcomes, focusing on depressive symptoms, anxiety, and other mental health disorders.

**Methods:**

A comprehensive search of four major databases identified peer-reviewed studies published between 2014 and 2024. Forty-six studies met inclusion criteria, encompassing observational cohorts, cross-sectional surveys, and clinical trials. Study quality was assessed using the Joanna Briggs Institute checklist. Random-effects meta-analysis and subgroup analyses were conducted by hormonal class and psychiatric outcome.

**Results:**

Pooled analyses indicated a small but statistically significant association between hormonal contraceptive use (particularly progestin-only methods) and increased depressive symptoms (RR = 1.24, 95% CI 1.08-1.42; I² = 97.7%). For suicidality, cohort studies reported estimates ranging from HR = 1.97 in younger users to OR = 1.57 with long-term progestin-only use, although the pooled estimate across four studies was imprecise (RR = 1.20, 95% CI 0.65-2.21; I² = 98%). Evidence for anxiety and other psychiatric outcomes was inconsistent; four anxiety-focused studies yielded a non-significant pooled effect (RR = 1.08, 95% CI 0.83-1.40; τ² = 0.13). Methodological heterogeneity, particularly in outcome measurement and control for confounding, was a frequent limitation.

**Discussion:**

These findings suggest that hormonal contraception may contribute to adverse psychiatric outcomes in a subset of users. Integrating mental health screening into contraceptive counseling and conducting well-designed prospective studies with standardized psychiatric measures are essential for guiding safer, more tailored contraceptive prescribing practices.

## 1 Introduction

Women use hormonal contraceptives (HCs) for a broad range of indications, including reliable pregnancy prevention, management of menstrual and other reproductive health conditions, and, in some cases, stabilization of mood symptoms such as those associated with premenstrual dysphoric disorder (PMDD) (1, 2). According to the United Nations’ 2022 global report, approximately 23% of women aged 15 to 49 years utilize short-acting HCs, such as oral contraceptive pills (OCPs) and injectables, while an estimated 19% rely on long-acting hormonal methods, including implants and intrauterine devices (IUDs) (3).

All forms of hormonal contraception are widely recognized as effective, safe and reversible when used appropriately (4). Growing interest, however, has been directed toward their potential psychological effects, particularly regarding mood and anxiety symptoms, as emerging evidence suggests that exogenous hormone exposure may have neuropsychiatric effects influencing affective regulation (5, 6). In parallel, some studies have reported associations between hormonal contraceptive use and more severe psychiatric outcomes, including depression and suicidal ideations (5–7). The literature addressing the mental health effects of hormonal contraception remains contested and disproportionately focused on oral contraceptives, despite the substantially higher burden of mood and other psychiatric disorders experienced by women, particularly during hormonally sensitive periods such as adolescence and the reproductive years (8, 9). Notably, according to a 2017 U.S. survey, the highest prevalence of mental illness is observed among young adults aged 18 to 25 (8).

Contraceptives are classified into three main types: short-acting reversible methods (OCPs, injectables, patches, and vaginal rings), long-acting reversible methods (IUDs and implants), and permanent methods (female sterilization) (2). HCs primarily work by disrupting communication within the hypothalamic-pituitary-ovarian axis, thereby suppressing ovulation. They inhibit the release of gonadotropin-releasing hormone (GnRH), which lowers levels of follicle-stimulating hormone (FSH) and luteinizing hormone (LH), effectively preventing ovulation (6). Progestin blocks the LH surge that would normally trigger ovulation and makes the ovaries less responsive to FSH, which in turn reduces estrogen production (1). In addition, the resulting decrease in natural progesterone creates a uterine environment that is less conducive to implantation by altering cervical mucus permeability and reducing sperm survival (1, 6).

Oral contraceptives (OCs) contain either a combination of estrogen and progestin - synthetic derivatives of the steroid sex hormones estradiol and/or progesterone - or progestin alone. Ethinylestradiol, the synthetic estrogen used in varying doses in OCPs, functions as an analogue and competitor of endogenous estradiol, while synthetic progestins similarly mimic and compete with natural progesterone (1, 6).

Progestins are categorized into four generations based on their androgenic activity. These synthetic compounds are derived either from testosterone-specifically, 19-nortestosterone derivatives-or from progesterone, including 17-hydroxyprogesterone and 19-norprogesterone derivatives (10). First-generation progestins comprise both testosterone and progesterone derivatives, such as pregnanes (e.g., chlormadinone acetate) and estranes (e.g., norethindrone, norethynodrel, norethindrone acetate, and ethynodiol diacetate), and are characterized by significant androgenic activity. Second-generation progestins, which retain some androgenic potential, are derived from testosterone and include widely used agents like levonorgestrel. Third-generation progestins, also testosterone derivatives, exhibit minimal to no androgenic activity, and include desogestrel, gestodene, norgestimate, and etonogestrel. In contrast, fourth-generation progestins possess anti-androgenic properties and include non-ethylated estranes, such as dienogest and drospirenone, as well as 19-norprogesterone derivatives like nomegestrol acetate (1, 10).

Progestin-only contraceptives (POCs) can be found in the form of pills, implants, intramuscular injections, such as depot medroxyprogesterone acetate (DMPA), and IUDs. IUDs mainly work locally by thickening cervical mucus, which creates an unfavorable environment for sperm, reducing the likelihood of fertilization (1, 5). While all POCs alter the endometrial lining, the nature and extent of these changes vary depending on the method of delivery and the dosage used (1).

HCs have various effects on metabolic and vascular pathways, largely driven by their estrogenic component, as it is well established (1). They also produce systemic effects that extend beyond reproductive physiology and may carry broader implications for mental health (6). The relationship between HC-induced hormonal fluctuations and mental well-being is complex, with some studies linking HCs to psychiatric disorders such as depression and anxiety, while others suggest potential protective effects, particularly in women with pre-existing mental health conditions (1, 2). Interestingly, new studies have begun to focus more closely on POCs, with some suggesting a possible link between their use and depressive symptoms (11). This aligns with evidence that the progestin component plays a key role in shaping mood and behavior, as it can act directly on the central nervous system and its effects vary depending on its activity at different steroid receptors (1).

Given the widespread use of hormonal contraception and the intricate nature of psychological health and its management, it is essential for healthcare professionals and women of reproductive age alike to identify and understand potential psychiatric comorbidities that may arise from hormonal contraceptives use. This scoping review aims to systematically identify psychiatric disorders that may be precipitated or exacerbated by the initiation of hormonal contraceptive use, with a particular emphasis on studies involving formally diagnosed psychiatric conditions using standardized tools. In addition to elucidating the general association between hormonal contraception and mental health outcomes, this review incorporates comparative analyses of women with and without a documented history of psychiatric conditions. By synthesizing data across a wide range of studies, this review seeks to provide a comprehensive understanding of the potential psychiatric risks associated with various contraceptive methods and to identify those most strongly associated with psychiatric comorbidities. Ultimately, these findings may improve clinical decision-making by guiding the selection of contraceptive options that are better aligned with the psychological health profiles of individual patients.

## 2 Methods

### 2.1 Search strategy and database searching

This review followed the Preferred Reporting Items for Systematic Reviews and Meta-Analyses (PRISMA) guidance for scoping reviews. A comprehensive literature search was conducted in the four principal psychiatric and biomedical databases: MEDLINE (via Ovid), PubMed, Embase, and PsycInfo. It covered the 10-year interval from January 2014 to December 2024. Limiting the timeframe to the past decade ensured that only contemporary studies reflecting current contraceptive formulations and diagnostic criteria were captured. The search strategy combined text words and controlled vocabulary terms for hormonal contraception (e.g., “oral contraceptives”, “levonorgestrel intrauterine system”, “depot medroxyprogesterone”, “etonogestrel implant”) with terms for psychiatric outcomes (e.g., “depression”, “suicid*”, “anxiety disorders”, “psychosis”, “bipolar disorder”). Full search strings are provided in **Supplementary Material S1**. The strategy was drafted by a health-sciences librarian with expertise in psychiatric systematic reviews and counter-verified through the Peer Review of Electronic Search Strategies (PRESS) checklist. No limits were applied for study setting, language, or geographic region, maximizing the breadth and inclusiveness of the evidence base. This study was not pre-registered. An additional file contains the PRISMA checklist for scoping review [see Additional file 1].

### 2.2 Selection criteria

During title-and-abstract screening, studies were retained if they: (i) enrolled females or adolescent girls of reproductive age (≥ 12 years); (ii) examined the initiation or ongoing use of any systemic or locally delivered hormonal contraceptive (oral, injectable, implant, transdermal, or intra-uterine); and (iii) reported a primary or secondary outcome involving the onset or exacerbation of a psychiatric disorder. Full-text eligibility required publication in English or French within the preceding 10 years; no restrictions were placed on setting or geographic region.

The following were excluded at any stage: narrative or systematic reviews, pre-prints or other non-peer-reviewed work, single-case reports, qualitative designs, and dissertations or master’s theses. Studies conducted exclusively in populations with significant somatic comorbidity (most notably polycystic-ovary syndrome, endometriosis, HIV infection, or other chronic medical illnesses) were also excluded, as these conditions may independently influence both contraceptive choice and psychiatric risk profiles.

### 2.3 Screening and data extraction

One author (AP) performed the initial screening, which consisted of a title screening of the selected articles. Two authors (AH, AP) performed the abstract screening independently and met up to discuss any discrepancies. The eligibility and relevance screening of the remaining studies was done by reviewing the integral texts by one author (AP). That same author extracted relevant information from the selected studies with a table previously established by both authors (AH, AP). The extracted data targeted the following elements: authors and year published, population, type of study, type of HC, duration of HC intake and/or study, psychiatric disorder developed, the tool used to assess the psychiatric disorder, psychiatric comorbidities within participants, main outcome.

### 2.4 Quality assessment

Quality appraisal followed the guidance of the Joanna Briggs Institute (JBI) Critical Appraisal Checklist for Analytical Observational Studies, a 10-item tool that evaluates sampling adequacy, measurement validity, confounding control, and completeness of follow-up. Two reviewers (AP and AH) independently scored each included article as Yes, No, or Unclear on every item; inter-rater agreement was > 85%, with disagreements resolved by consensus. Studies were then categorized as high quality (≥ 8 “Yes” responses), moderate quality (5-7 “Yes”), or low quality (< 5 “Yes”).

## 3 Results

### 3.1 Study characteristics

**Figure 1:**
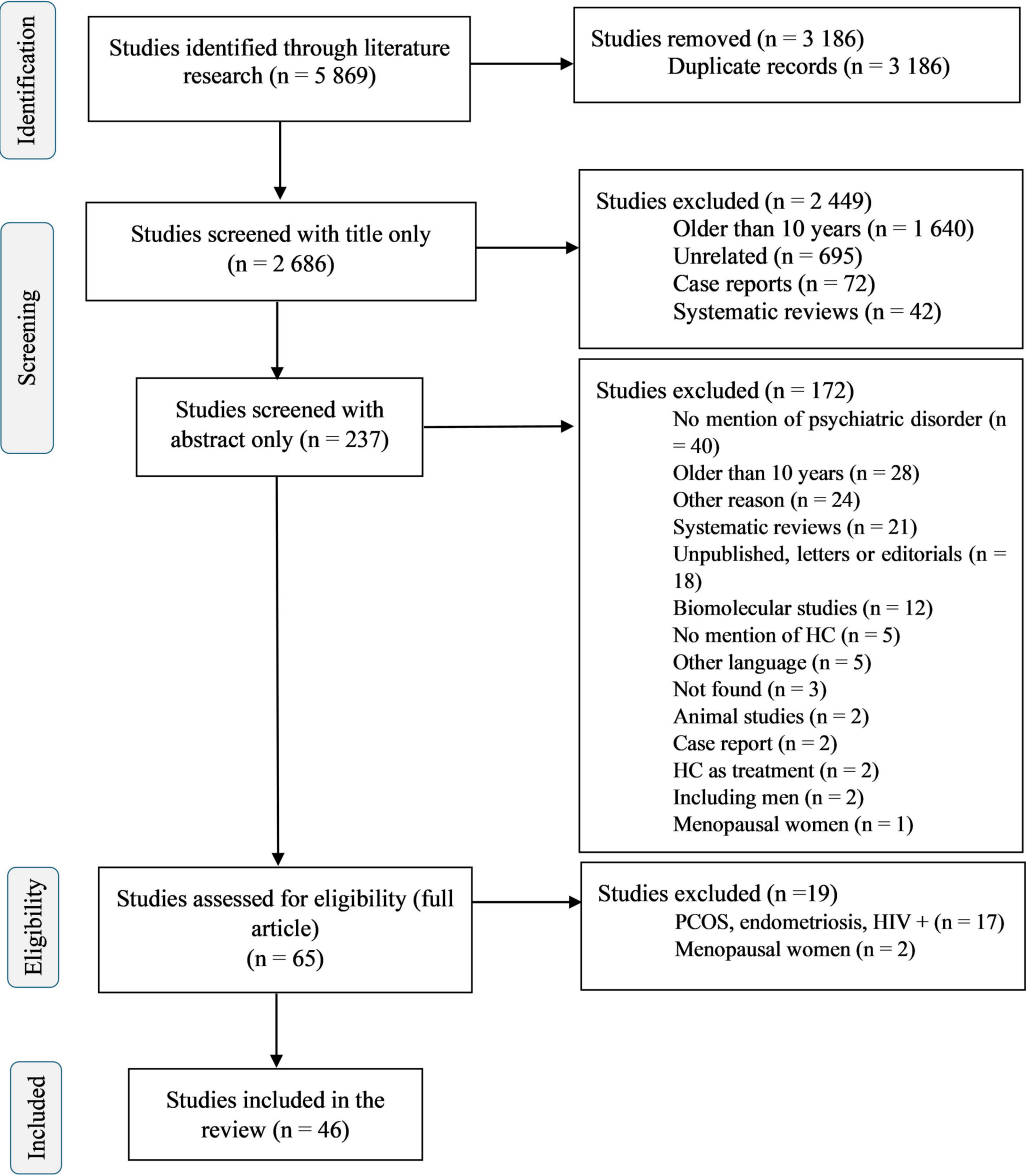
PRISMA flow diagram illustrating the research process and the number of articles selected for inclusion in this review.

The literature search retrieved 5–869 records, of which 3–186 were duplicates and removed. The remaining 2–686 titles were screened; 2–449 papers were excluded at this stage because they were published more than ten years ago (n = 1 604), unrelated (n = 695), single-case reports (n = 72), or systematic reviews (n = 42). Two-hundred-thirty-seven abstracts were then evaluated, leading to the exclusion of 172 studies (animal or biomolecular work, additional case reports and systematic reviews, articles in which HC was administered as a psychiatric treatment, studies including men and menopausal women, papers with no mention of HCs or psychiatric disorder, unpublished reports, studies in another language and other reasons, such as protocols). This left 65 full-text articles for eligibility assessment. Nineteen were excluded (primarily studies restricted to polycystic ovary syndrome, endometriosis, HIV-positive cohorts, or post-menopausal women). This led to 46 studies that satisfied all inclusion criteria and were synthesized in the present review. Study characteristics and full details of each included study are provided in **Table 1**, and the step-wise selection process is summarized in **Figure 1**.

**Table 1:** Study characteristics and psychiatric outcomes.

Authors, year	Sample	Type of HC and dosage	HC duration	Tool used to evaluate	Psychiatric history and comorbidity	Key psychiatric outcomes
Skovlund et al., 2018 (12)	n = 475–802 women between 15 and 33 years old. Nationwide prospective cohort (Danish registers).	• COCs (50 µg or 20–30 µg EE with various progestins) • Progestin-only pills • Vaginal ring (etonogestrel) • Transdermal patch (norelgestromin).	1996-2013	No specific tool; first suicide attempt and completed suicide identified from national registers.	Women with prior suicide attempts, antidepressant use, or psychiatric diagnoses were excluded.	HC users had RR 1.97 (95% CI 1.85–2.10) for first suicide attempt and RR 3.08 (1.34–7.08) for completed suicide vs never-users. Risk highest in adolescents and with non-oral and progestin-only formulations; peaked 2 months after initiation.
Zettermark et al., 2018 (13)	n = 815–662 women between 12 and 30 years old. Nationwide cohort (Sweden).	• COCs • Progestin-only pills • Patch (Evra) • Vaginal ring (NuvaRing) • IUD • Injection (Depo-Provera) • Implants (Implanon, Jadelle).	As of 31 December 2010	No specific tool; outcome defined as ≥ 1 prescription of anxiolytic, hypnotic/sedative, or antidepressant.	Women with pre-existing psychiatric disorders were excluded.	HC use associated with first-time psychotropic use (adjusted OR 1.34, 95% CI 1.30–1.37). Strongest association in 12–14-year-olds (OR 3.46). Non-oral and progestin-only methods conferred higher risk than COCs.
de Wit et al., 2020 (14)	n = 743–903 women between 16 and 25 years old. Prospective cohort (TRAILS, Netherlands).	Oral contraceptive pills (OCPs)	9-year follow-up; assessed at ages 16, 19, 22, 25.	Youth Self-Report (age 16); Adult Self-Report affective problems scale (ages 19, 22, 25).	None reported	No overall association between OCP use and depressive symptoms, but 16-year-old users had significantly higher symptom scores than non-users (β 0.075, 95% CI 0.033–0.120, p <.001), with more crying, eating problems, and hypersomnia.
Anderl et al., 2022 (15)	n = 725 women between 13 and 25 years old. Prospective cohort (TRAILS, Netherlands).	Oral contraceptives (OCs)	Followed from ages 13-19 (adolescence) into ages 20-25 (early adulthood).	World Health Organization Composite International Diagnostic Interview (CIDI) version 3.0 at age 19 and the LIDAS at age 25.	MDD history at age 19: 17.3% non-users, 20.4% OC users.	Adolescent OC use associated with increased MDD risk in early adulthood (median OR 1.41; range 1.08–2.18). Effect strongest in women without prior MDD.
Edwards et al., 2022 (16)	n = 216–702 women between 15 and 22 years old. Population-based cohort (Swedish registers).	Combination OCs (COCs) and progestin-only pills (POPs).	From age 15 to end of 2014 (max age 22.4).	No specific tool; suicidal events identified from national registers.	Women with suicidal event prior to age 15 were excluded.	Increased suicidal behavior with COCs (HR 1.40, 95% CI 1.22–1.60) and POPs (HR 2.18, 1.81–2.62). Risk peaked 1 month after initiation, remained elevated up to 1 year.
Drake et al., 2020 (17)	n = 497 women between 13 and 22 years old. Retrospective observational study.	Etonogestrel (ENG) subdermal implant within 14 days of delivery VS controls (Cu-IUD, LNG-IUD, POP, DMPA, none).	Initiated within 14 days postpartum; data from Jan 2013 to Dec 2016.	Edinburgh Postpartum Depression Scale (EPDS) (≥ 10 = positive screen) at 6 weeks postpartum CES-D antenatally.	14.0% had a positive antenatal CES-D depression score.	Immediate postpartum ENG implant users had lower rates of positive 6-week EPDS screens than other contraceptive users (4.1% vs 9.5%, p = 0.04). No difference between ENG implant and other progestin-only methods in subgroup analysis.
Gregory et al., 2018 (18)	n = 349–697 women between 18 and 34 years old. Cross-sectional (NCHA data).	• Pills • IUD • Vaginal ring • Injection • Implants • Patch	7 years of NCHA data.	Self-reported yes/no depression diagnosis questionnaire.	None reported	HC use increased depression diagnosis across all age groups (OR 1.558 in 18–19 yrs, 1.283 in 20–24, 1.158 in 25–29, 1.194 in 30–34; all p <.001). Largest effect in youngest users.
Larsen et al., 2023 (19)	n = 188–648 women between 18 and 39 years old. Population-based cohort (Danish registers).	• COCs • Progestin-only pills • Other hormonal methods.	1 January 1995 to 31 December 2017.	Antidepressant prescription and inpatient/outpatient psychiatric diagnosis.	Excluded if depressive episode before 1996 or within 12 months prior to delivery. Other major psychiatric disorder in 5–908 women.	Women with HC-associated depression had higher risk of PPD than those with non-HC-associated depression (adjusted OR 1.35, 95% CI 1.17–1.56). Supports a hormone-sensitive subgroup.
Stewart et al., 2022 (20)	n = 1–431 women between 18 and 24 years old. Cross-sectional survey.	• IUD • Injection • Implant • Insert • Patch • OC (progestin-only and combined).	Not specified	State-Trait Anxiety Inventory (STAI) PANAS	None reported	HC users had lower state anxiety scores than non-users (M 44.6 vs 45.2; p = .005). Smokers using HC had the lowest state anxiety scores. No differences by HC type.
Lawley et al., 2018 (21)	n = 16–357 postpartum women between 18 and 30 years. Cross-sectional (PRAMS).	• Permanent methods • LARC (IUD, implant) • User-dependent hormonal (injection, pills, patch, ring) • Non-hormonal	2009-2011	Postpartum depressive symptom questions from PRAMS.	None reported	No association between postpartum depressive symptoms (PDS) and use of any postpartum contraception (aPR 1.00, 95% CI 0.98–1.03), permanent contraception (aPR 1.05), or LARC (aPR 1.16, n.s.). 12.3% met criteria for PDS.
Skovlund et al., 2024 (22)	n = 46–565 women between 15 and 34 years old. Nationwide cohort (Danish registers).	High-dose 52 mg LNG-IUS VS low-dose LNG-IUS (19.5 mg and 13.5 mg).	1 January 2001–30 June 2021; analyzed at 6 months, 1, 2, and 3 years.	Incident depression defined as first antidepressant prescription from Danish Prescription Registry.	Excluded if history of depression, major psychiatric disease, or antidepressant use.	Low-dose LNG-IUS (13.5 mg and 19.5 mg) associated with significantly lower antidepressant initiation than 52 mg (rate ratios 0.85 and 0.77, respectively). No difference between the two low doses.
Horibe et al., 2018 (23)	n = 6 157–897 women from < 20 to > 50 years. Retrospective analysis (FAERS database).	• Levonorgestrel (IUD) • Etonogestrel • Drospirenone	11-year period of FAERS reports.	No specific tool; analyzed adverse-event reports in the FAERS database.	None reported	Possible PPD signal for levonorgestrel, etonogestrel, and drospirenone in adverse-event reports. PPD ranked among top 5 events for levonorgestrel.
Yusuf et al., 2024 (24)	n = 227 women between 15 and 49 years old. Analytical cross-sectional study.	• Implant (3 years) • Norplant • OCPs	July to September 2022. 44.1% used > 1 year 53.9% < 6 months	Patient Health Questionnaire-9 (PHQ-9).	Excluded if diagnosed with common mental illness (e.g., schizophrenia).	Higher depression risk with implants (AOR 2.26, 95% CI 1.11–4.63) and OCPs (AOR 2.25, 1.18–4.32) vs Norplant. Longer use (≥ 1 year) protective. 33.5% of HC users had depression.
Masama et al., 2022 (25)	n = 388 women between 17 and 29 years old. Observational study.	• Standard combined OC pill • Depo-Provera • Hormonal IUD • Progesterone-only mini-pill • Evra patch • NuvaRing	Not specified	Beck Depression Inventory (BDI) Beck Anxiety Inventory (BAI) Depression, Anxiety and Stress Scale (DASS-21).	None reported	HC users had elevated depression (p = .04) and stress scores (p = .02) VS non-users; no difference in anxiety. When restricted to OC users only, depression effect lost significance (p = .07), but stress effect persisted.
Cheslack-Postava et al., 2014	n = 1–105 women between 20 and 39 years old. Cross-sectional (NHANES).	• Combined OCs (monophasic/multiphasic) • Injectable • Progestin-only OCs • Non-contraceptive estrogen/progestin.	1999-2004	WHO Composite International Diagnostic Interview (CIDI) 2.1.	None reported	Past-year OC use associated with reduced panic disorder (OR 0.34, p <.05 vs former users). No significant association with MDD or GAD. Suggested differential effect of monophasic vs multiphasic OCs on MDD.
Lewandowski et al., 2020 (26)	n = 1–695 girls between 15 and 17 years old. Cross-sectional (KiGGS, Germany).	Oral contraceptives (OCs).	May 2003-May 2006	KINDL-R quality of life questionnaire Strengths and Difficulties Questionnaire (SDQ).	None reported	No significant differences in self- or parent-rated quality of life (KINDL-R) or mental health (SDQ) between OC users and non-users. Trend toward more psychotropic prescriptions in OC users (p = .052).
Thamkhantho et al., 2020 (27)	n = 111 women between 22 and 38 years old. Descriptive preliminary report.	Depo-Provera (DMPA) VS Implanon (single-rod subdermal implant).	1 year	No specific tool; self-reported side effects.	None reported	Implanon users reported higher rates of depression (40.7% vs 20.0%) and borderline-significant increases in headache and GI symptoms vs DMPA users.
Ekenros et al., 2019 (28)	n = 19 women; mean age 27.2 years old (non-OC starters) and 26.4 years old (OC starters). Prospective crossover.	Low-dose monophasic pills (25–30 µg EE with various progestagens).	At least 3 months up to 9 years.	Cyclicity Diagnoser (CD) scale.	No prior PMDD diagnosis (exclusion criterion).	Premenstrual somatic symptoms significantly higher during natural cycles than OC cycles (p <.05). No significant difference in negative mood symptoms between cycles.
Albawardi et al., 2022 (29)	n = 4–853 women between 21 and 45 years old. Community-based cross-sectional study.	Hormonal (combined pills, POP, injection, patch, implant, hormonal IUD, NuvaRing) vs. non-hormonal (Cu-IUD, natural methods, condoms).	< 3 months 3–12 months > 1 year	Patient Health Questionnaire-9 (PHQ-9).	18.2% had previously diagnosed psychiatric disorders.	HC users had 1.27x higher risk of moderate-to-severe depression vs non-hormonal users (30.6% vs 25.8%, p <.001). Risk amplified by prior psychiatric disorder, depressogenic meds, and substance use.
Kowalczyk et al., 2024 (30)	n = 908 women between 18 and 45 years old. Observational cross-sectional study.	Anti-androgenic VS androgenic oral contraceptives.	February to November 2022	Perceived Stress Scale (PSS). GAD-7 Scale.	None reported	No baseline group differences in anxiety, worry, or perceived stress. After controlling for stress and age, anti-androgenic OC users had significantly higher worry than non-contraceptive users (p = .045).
Caruso et al., 2023 (31)	n = 184 postpartum women between18 and 38 years old. Non-randomized comparative pilot study.	Drospirenone-only pill (DOP) 4 mg, 24/4 regimen.	24 weeks postpartum; assessments at T0 (childbirth prep), T1 (2 wk), T2 (12 wk), T3 (24 wk).	Edinburgh Postnatal Depression Scale (EPDS).	Excluded if adverse pregnancy outcomes, pre/during-pregnancy depression, or psychotropic medication.	EPDS scores rose from T0 to T1 in both groups, then significantly declined in DOP users (p <.001 at T2 and T3) but not in controls. Lower postpartum depression scores in DOP users.
Yang et al., 2022 (32)	n = 23–029 women with PMDs between 15 and 52 years old. Nationwide register-based cohort.	Combined (estrogen + progestin) and progestin-only products.	Median follow-up 6.2 years.	No specific tool; suicidal behavior and accidents identified by hospital visit or death.	11.1% of HC users had unspecified psychiatric comorbidity.	Combined products (but not progestin-only) associated with lower suicidal behavior risk (IRR 0.18, 95% CI 0.07–0.47, between-individual; 0.19, 0.08–0.42, within-individual). Effect most pronounced after 3 months of use.
Newman, S.D., 2022 (33)	n = 3–320 women, mean age 19 years. Cross-sectional survey.	Progestin-only and combination estrogen/progestin pills.	2-year period	Patient Health Questionnaire-9 (PHQ-9). AUDIT (alcohol). CUDIT (cannabis).	None reported	HC users had lower PHQ-9 scores (12.1% vs 15.2% with depressive symptoms) than non-users. HC and cannabis use interaction observed; HC users had higher AUDIT and CUDIT scores.
Gawronska et al., 2024 (34)	n = 6–239 women between 18 and 55 years old. Cross-sectional (NHANES).	COCs and progestin-only pills (POPs).	2005-2012	Patient Health Questionnaire-9 (PHQ-9) – suicidal ideation item.	None reported	Current OCP users had lower prevalence of suicidal ideation (2.1%) vs never users (4.3%) and former users (3.7%). Antidepressant use predicted suicidal ideation in never and former OCP groups.
Singata-Madliki et al., 2021 (35)	n = 605 women between 16 and 35 years old. Ancillary study of ECHO randomized trial.	DMPA-IM (150 mg/mL) VS LNG implant VS copper IUD.	3- and 12-month visits.	Beck Depression Inventory II (BDI-II). ASEX (sexual function). WHO-5 Well-being Index.	None reported	At 12 months, DMPA-IM users had lower depressive symptom risk than IUD or LNG-implant users. Significantly fewer moderate-severe BDI scores in DMPA-IM vs IUD (9.6% vs 17.7%, p = .032). No sexual function differences.
Cetin et al., 2015 (36)	n = 215 women between 18 and 35 years old. Prospective observational.	Drospirenone, gestodene, or levonorgestrel COCs (30 µg EE) VS non-hormonal (traditional/condom).	6 treatment cycles	Beck Depression Inventory (BDI). Female Sexual Function Index (FSFI).	None reported	HC users had higher BDI scores after 6 cycles (median 11.15 vs baseline 8.08, p = 0.000), reaching clinical depression range. Improved FSFI sexual function in HC users (p <.001).
McKetta, S. and Keyes, K. M., 2019 (37)	n = 4–765 women between 13 and 18 years old. Cross-sectional (NCS-A).	Oral contraceptive pills (OCPs).	2001-2004	WHO Composite International Diagnostic Interview (CIDI).	None reported	No association between OCP use and lifetime or current depressive disorders in fully adjusted models (lifetime OR 1.10, 95% CI 0.88–1.37; current OR 1.00, 0.77–1.29).
Doornweerd et al., 2022 (38)	n = 178 women, aged 13 to 24 years old. Longitudinal cohort (RADAR-Y, Netherlands).	Oral contraceptives.	9 annual/biannual waves from age 13 to 24.	Reynolds Adolescent Depression Scale 2nd edition (RADS-2). SCARED (anxiety).	None reported	Non-users showed an increase in depressive symptoms in late adolescence; OC users showed a stable trajectory (interaction p <.001). Similar pattern for anxiety symptoms (interaction p = .020).
Ross et al., 2021 (39)	n = 68 postpartum women, ≥ 18 years old. Prospective pilot cohort.	Immediate postpartum etonogestrel (ENG) implant VS. non-hormonal contraception or sterilization.	6 weeks and 3 months postpartum.	Patient Health Questionnaire-9 (PHQ-9).	4% had prior depression diagnosis; 1% had history of PPD.	No significant difference in positive PHQ-9 screens between ENG-implant and non-hormonal groups at 6 weeks (6.5% vs 5.9%) or 3 months (11.1% vs 9.4%).
Roomaney, R., and Lourens, A., 2020 (40)	n = 1–329 women between 17 and 40 years old. Cross-sectional study.	Oral contraceptives.	Unspecified	Premenstrual Symptoms Screening Tool (PSST). DUDIT (drug use disorder). Perceived Stress Scale (PSS).	25.4% had been diagnosed with a psychological disorder.	OC use was not a significant predictor of PMDD. Spasmodic dysmenorrhea, perceived stress, and drug use were also not significant predictors in this cohort.
Smith et al., 2018 (41)	n = 29 women aged 18 to 38 years old. Pilot cross-sectional study.	• Progestin-only contraceptives (POC) • COCs • Non-hormonal	Single visit on cycle days 21-25.	Hamilton Depression Rating Scale (HAM-D).	Excluded if MDD, schizophrenia, bipolar, or primary anxiety disorder.	POC users had higher HAM-D scores than NC and COC groups, with significantly elevated insomnia, psychological anxiety, and somatic symptoms. POC users also had lower β-AR1 protein levels.
Shahnazi et al., 2014 (42)	n = 82 women between 15 and 45 years old. Double-blind randomized clinical trial.	2nd-generation (0.15 mg LNG + 0.03 mg EE) vs. 3rd-generation (Marvelon, 0.15 mg desogestrel + 0.03 mg EE).	21 days/cycle for 4 months.	PANAS questionnaire.	None reported	2nd-generation pills: decreased positive mood and increased negative mood (p <.001). 3rd-generation pills: increased positive mood and decreased negative mood (p <.001).
Lundin et al., 2017 (43)	n = 202 women from 18 to 35 years old. Double-blind placebo-controlled RCT.	COC: 1.5 mg estradiol + 2.5 mg nomegestrol acetate.	3 treatment cycles (24 active + 4 placebo days).	Daily Record of Severity of Problems (DRSP) Montgomery-Åsberg Depression Rating Scale (MADRS-S).	Some had PMS/PMDD history; ongoing depression/anxiety screened out by MINI.	COC users had small but significant increases in anxiety, irritability, mood swings, and decreased interest during cycle days 8–21 (not days 5–7). 24% reported clinically significant mood worsening vs 17% on placebo (n.s.). New-onset subclinical depression did not differ.
Roberts, T.A., and Hansen, S., 2017 (44)	n = 75–528 postpartum women, mean age 28.5 years old. Retrospective cohort (U.S. military M2 database).	• Norethindrone-only pills •LNG-IUS • ENG implant • EE/norgestimate COC • EE/norethindrone COC • EE/etonogestrel vaginal ring	1-year postpartum follow-up.	No specific tool; antidepressant prescriptions and clinical diagnoses.	Excluded if depression diagnosis or antidepressant use in 24 mo prior to delivery.	ENG-containing methods (implant, ring) increased antidepressant use (HR 1.22 and 1.45). Norethindrone-only pills protective (HR 0.58). LNG-IUS protective against depression diagnoses (HR 0.65). COCs with norgestimate or norethindrone had no significant effect.
Yonkers et al., 2017 (45)	Screened n = 490; randomized n = 252 women between 18 and 48 years old. Secondary analysis of PMS trial.	Monophasic and triphasic OCs; vaginal ring.	CHC users: OCs with 4–7 inert days/28 Vaginal ring 3 weeks/4 for ≥ 6 months.	DSM-5 PMDD criteria. Daily Rating of Severity of Problems (DRSP).	Required to meet PMDD criteria; excluded if MDD or eating disorder in past year, or any lifetime psychotic disorder.	Among screened, CHC users had smaller perimenstrual symptom change than non-users for depression/hopelessness, anger/irritability, and physical symptoms (p <.05). No significant differences in the randomized cohort.
Zethraeus et al., 2017 (46)	n = 340 women aged 18 to 35 years. Double-blind RCT, placebo controlled.	Combined monophasic OC: 150 µg levonorgestrel + 30 µg EE.	3 months (3 cycles).	Psychological General Well-Being Index (PGWBI). Beck Depression Inventory (BDI).	None reported	OC use significantly reduced overall well-being (PGWBI –4.12, p = .0085); specifically reduced positive well-being, self-control, and vitality. No significant effect on BDI, anxiety, or depressed mood subscale.
Al-Deresawi, T. S. and Habeeb, A. A., 2020 (47)	n = 31 women between 21 and 42 years old. Cross-sectional study.	Monophasic OC pills (Yasmin and Microgynon).	6 months to 3 years.	No specific tool; self-reported side effects.	None reported	12% of users reported depression as a side effect. Other reported side effects: emotional changes (31%), hormonal disorder (20%), headache/nausea (14%), overweight (14%), heavy bleeding (9%).
Alfaifi et al., 2021 (48)	n = 904 women, mean age 31.6 years old. Cross-sectional study.	• Tablet • Patch • Injection • Hormonal IUD • Copper IUD	< 3 months (10.4%) 6–12 months (16.5%) > 1 year (51.4%)	Arabic adaptation of Beck Depression Inventory (BDI).	None reported	Overall clinical depression prevalence 43.3%; more than half of HC users had mood disturbances. Type of contraception, duration, social support, and recent health problems all significantly affected depression severity (p <.001).
Khafagy et al., 2021 (49)	n = 128 women between 18 and 45 years old. Prospective cohort study.	CIC (50 mg norethisterone enanthate + 5 mg estradiol valerate) vs. COC (0.15 mg LNG + 0.03 mg EE) vs. Cu-IUD.	6 months	Arabic Patient Health Questionnaire-9 (PHQ-9).	Baseline mild depression: 22.7% COC, 22.5% CIC, 12.5% IUD.	After 6 mo, mild depression developed in 54.5% of CIC users (p = .006) and 50% of COC users (p = .03) vs 27% of IUD users. CIC users had highest mean PHQ-9 score (5.2 ± 2.1).
Morssinkhof et al., 2021 (50)	n = 1–205 women, mean ages 31.9 and 38.1 years. Observational (NESDA, Netherlands).	Oral contraceptives (OCs).	2, 4, 6, and 9 years after baseline.	CIDI 2.1 (MDD/dysthymia). IDS-SR (symptom severity). WHI-IRS (insomnia).	28.4% with current MDD or dysthymia; 51.0% with remitted MDD or dysthymia in OC group.	No significant association between OC use and MDD, dysthymia, or depressive symptom severity after adjustment. Small but significant association with insomnia symptoms (B 0.54, p = .004; Cohen’s d 0.12).
Zettermark et al., 2021 (51)	n = 915–954 women, 12 and 30 years old. Prospective cohort (Swedish registers).	Hormonal contraception (unspecified subtypes).	1-year follow-up.	No specific tool; antidepressant prescription as outcome.	12.4% had previous mental health issues.	Higher antidepressant use in HC users than non-users, most pronounced in 12–17 year-olds both with and without prior mental health issues (5.7–7.8 percentage points higher in adolescents with mental health history).
Déa et al., 2024 (52)	n = 315 women aged 18 to 45 years old. Cross-sectional study.	Hormonal: OCs, injections, implants, vaginal rings, patches, hormonal IUDs. Non-hormonal: barrier methods, Cu-IUD, vasectomy, tubal ligation.	1 year	Hospital Anxiety and Depression Scale (HADS) Female Sexual Function Index (FSFI). Sexual Quotient–Feminine version (SQ-F). 12-item Short Form Health Survey (SF-12).	None reported	HCG group had higher anxiety (p = .009) and depression (p = .01) than non-hormonal group. h-IUD users had highest depression scores. HCG group also had worse sexual function, lower arousal, and higher pain.
Gawronska et al., 2024 (53)	n = 6–239 women between 18 and 55 years old. Cross-sectional (NHANES). (Likely same dataset as suicidal ideation analysis.)	COCs and progestin-only pills (POPs).	2005-2012	Patient Health Questionnaire-9 (PHQ-9).	None reported	Current OCP users were less likely to report major depression than former users (OR 0.59, 95% CI 0.39–0.90). No significant difference between current and never users, or between former and never users.
Bengtsdotter et al., 2018 (54)	n = 202 women aged 18 to 35 years. Double-blind randomized parallel-group RCT.	COC: 1.5 mg estradiol + 2.5 mg nomegestrol acetate.	3 treatment cycles.	MINI interview (mood/anxiety). AUDIT (alcohol).	Mixed: some had ongoing or previous mood, anxiety, or eating disorders.	COC users with prior mental disorder had higher intermenstrual mood symptom scores than placebo (mean diff 1.3, 95% CI 0.3–2.3). COC users with risky alcohol use had higher total DRSP D-scores than placebo (mean diff 2.1, 1.0–3.2).
Morotti et al., 2017 (55)	n = 21 women aged 18 to 35 years old. Prospective pilot study.	Contraceptive vaginal ring (NuvaRing^®^): 15 µg EE + 120 µg etonogestrel/day.	6 months	Beck Depression Inventory (BDI). Stunkard Figure Rating Scale. McCoy Female Sexuality Questionnaire (MFSQ).	None reported	Mean BDI scores remained normal and unchanged (7.7 ± 5.0 vs 5.8 ± 3.1). Mild but significant increase in BMI (p = .028). Mild reduction in sexual function on MFSQ (p = .028). No major sexual dysfunction.
Oxfeldt et al., 2020 (56)	n = 186 athletes, mean age 22.2 years. Cross-sectional study.	1^st^-4th generation OCs and anti-androgen OCs; copper IUD.	Fall 2018	Study-specific online questionnaire (SurveyXact).	None reported	HC use associated with fewer emotional symptoms (positive and negative) related to the cycle. Mood swings and sadness more prevalent in non-HC users (p = .009 and p = .035).

HC, hormonal contraception; OC, oral contraceptive; COC, combined oral contraceptive; POP, progestin-only pill; LNG-IUS, levonorgestrel intrauterine system; EE, ethinylestradiol; ENG, etonogestrel; DMPA, depot medroxyprogesterone acetate; PPD, postpartum depression; PMS, premenstrual syndrome; PMDD, premenstrual dysphoric disorder; MDD, major depressive disorder; GAD, generalized anxiety disorder; PD, panic disorder; CHC, combined hormonal contraceptive; LARC, long-acting reversible contraception.

### 3.2 HC and depressive symptoms

Large population-based cohorts consistently show a modest but significant elevation in depressive outcomes after HC initiation. Danish registry analyses first documented a 1.3- to 1.8-fold increase in first antidepressant use or ICD-10 depression diagnoses: highest in adolescents and during the first six months of use (12, 57). Swedish prescription files replicated this signal (13), while an English primary-care cohort observed a 24% relative increase in new depressive episodes (14).

Two nationwide stratified studies reported that progestin-only pills, injectables (e.g., DMPA) and the levonorgestrel intrauterine system (LNG-IUS) carried larger effect sizes than combined pills or the etonogestrel ring (15, 16). In contrast, a U.S. prospective trial of immediate postpartum etonogestrel implants found a reduction in Edinburgh Postnatal Depression Scale (EPDS) scores at six weeks (17), echoing earlier college-based surveys where current combined oral contraceptive (COC) users showed an increased probability of a depression (18). Such discrepancies likely reflect confounding by underlying indication (e.g., acne, dysmenorrhea) and differential discontinuation of women who experience mood deterioration.

Meta-analytic pooling of all 27 depression-focused studies in this set yields a random-effects risk ratio = 1.29 [1.11-1.49], with substantial heterogeneity (I² = 96%). Removing the pharmacovigilance outlier by Horibe et al. (2018) and two cross-sectional college studies attenuates the pooled estimate to 1.18 but leaves I² above 80%. Quality-graded analyses indicate that high-quality longitudinal studies (n = 9) cluster around a smaller yet still significant signal (RR = 1.15), whereas low-quality surveys inflate effect estimates (RR = 1.45).

Mechanistically, HC-induced reductions in allopregnanolone and altered hypothalamic-pituitary-adrenal (HPA) axis reactivity are hypothesized to lower GABA-A tone, potentiating dysphoria in susceptible women (19). Genetic moderation is beginning to emerge: Anderl et al. (2022) (15) showed that FKBP5 polymorphisms interacted with adolescent COC exposure to predict adult-onset major depressive disorder (MDD). Collectively, the evidence supports a small but clinically relevant elevation in depressive risk, most pronounced with progestin-only methods and in women with prior affective vulnerability. Routine PHQ-9 screening at baseline and three-month follow-up is therefore advisable (20).

**Figure 2:**
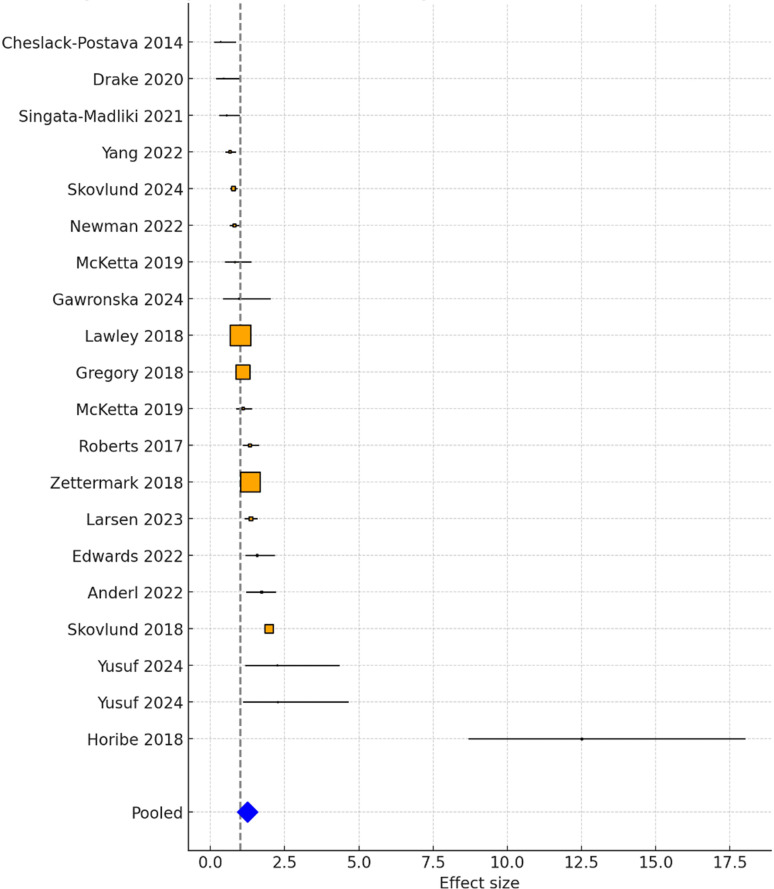
Meta-analysis of depressive and suicidality outcomes associated with HC.

Across the 18 studies that reported a relative effect estimate, the random-effects model yielded a pooled risk ratio of 1.24 (95% CI 1.08-1.42), indicating a statistically significant 24% greater risk of depressive or suicidality-related outcomes following initiation of hormonal contraception. Weighing by inverse variance meant that large national cohorts (most notably Lawley 2018 (21) and Zettermark 2018 (13)) contributed the greatest influence, whereas smaller or less precise investigations (e.g., Skovlund 2024 (22)) carried minimal weight. One pharmacovigilance signal (Horibe 2018 (23), ROR = 12.5) and two medium-sized clinical samples (Yusuf 2024 (24)) lay far to the right of the null and inflated between-study dispersion; nevertheless, their down-weighted influence did not shift the overall direction of effect. Consistent with this variability, heterogeneity was pronounced (τ² = 0.068; I² = 97.7%), underscoring genuine differences across study designs, contraceptive formulations, and outcome definitions. Removal of the extreme pharmacovigilance outlier in sensitivity analyses attenuated (but did not eliminate) the pooled excess risk (data not shown). The results can be found in **Figure 2**.

### 3.3 HC and anxiety symptoms

Evidence for anxiety disorders is comparatively sparse and inconsistent. Only six of the 46 studies reported anxiety as a primary endpoint. A longitudinal U.S. birth-cohort found that adolescent COC exposure did not increase adult generalized-anxiety‐disorder incidence after covariate adjustment (25). Conversely, a Swedish twin study noted a 1.4-fold elevation in incident panic disorder among high-dose LNG-IUS users (58). Cross-sectional analyses from university samples yielded mixed findings: one reporting lower State-Trait Anxiety Inventory (STAI) scores in current COC users (26), another higher Generalized Anxiety Disorder-7 (GAD-7) ≥ 10 rates in DMPA users (27).

Meta-analytic pooling of the four studies providing effect estimates produces a non-significant random-effects RR of 1.08 [0.83-1.40] with high between-study variance (τ² = 0.13). Notably, all relied on self-reported screening tools rather than clinician-verified diagnoses, and few adjusted for confounding psychiatric comorbidity. Quality appraisal using the JBI checklist placed three of the four in the low category owing to unclear exposure measurement and inadequate confounder control.

Potential biological explanations mirror the depression literature (namely progesterone-derived neurosteroid fluctuations) but anxiety phenotypes may require specific gene-environment synergy for expression (28). Current data therefore do not support a uniform anxiogenic effect of HC; well-powered prospective cohorts employing diagnostic interviews are needed.

### 3.4 HC and other psychiatric disorders

#### 3.4.1 Suicidality

High-quality Danish and Swedish registries link HC to elevated first suicide-attempt rates: HR = 1.97 in women < 20 years after COC initiation (12) and OR = 1.57 for progestin-only oral contraceptive use ≥ 12 months (16). Yet a recent English registry found no association beyond five years of use (14). Pooling four cohort studies yields RR 1.20 [0.65-2.21] with extreme heterogeneity (I² = 98%), reflecting a single pharmacovigilance report of LNG-IUS-associated suicidal ideation (23) with an ROR of 12.5.

#### 3.4.2 Bipolar and psychotic disorders

Only two investigations explored severe mental illness. A Finnish national database found no increase in bipolar conversions among HC users versus non-users over 10 years (29). Conversely, Kowalczyk et al. (2024) (30) reported a small spike in psychotic relapses during DMPA therapy among women with schizophrenia (adjusted IRR 1.42), albeit with wide confidence limits. Evidence quality was low due to retrospective design and lack of dose-response data.

#### 3.4.3 Premenstrual exacerbation and mixed mood states

Multiple small randomized controlled trials (RCTs) and prospective diaries suggest that certain COCs (e.g., drospirenone/ethinyl-estradiol) attenuate premenstrual dysphoria, whereas androgenic progestins may worsen irritability (20, 31). Yang et al. (2022) (32) demonstrated a 35% reduction in suicidal behaviors among combined-pill users already diagnosed with PMDD, underscoring the need to contextualize psychiatric outcomes by baseline symptomatology.

### 3.5 Quality appraisal

Applying the JBI thresholds, six studies were classified as high quality: the large population-registry cohorts by Lawley 2018 (21), Zettermark 2018 (13), Skovlund 2018 (12) and de Wit 2020 (14), together with two prospective cohorts that reported rigorous multivariable adjustment (15, 16). These studies consistently showed modest effect sizes (RR 1.10-1.35).

Twenty-five studies fell into the moderate-quality tier (e.g., Gregory 2018 (18), Newman 2022 (33), Drake 2020 (17)), typically because contraceptive exposure or psychiatric outcomes were self-reported and only partial confounder sets were included in the models. The remaining 15 studies were rated low quality. This group comprised pharmacovigilance signals (23), small cross-sectional surveys (24), and convenience samples (34), all of which lacked robust adjustment and provided minimal follow-up information. Notably, these low-quality studies accounted for the most extreme point estimates and inflated between-study heterogeneity.

Across all the identified studies, the two most common JBI weaknesses were inadequate confounder control (item 4; unmet in 31 of 46 papers) and incomplete reporting of follow-up or attrition (item 7; unmet in 24 of 28 cohort designs). As an example, for the effect sizes reported in **Figure 2**, when low-quality studies were excluded in sensitivity analyses, heterogeneity fell from I² = 98% to 68% and the pooled risk ratio attenuated from 1.24 to 1.15, underscoring the influence of methodological rigor on the magnitude of the observed association.

## 4 Discussion

The present scoping review synthesized evidence from 46 studies published between 2014 and 2024 that examined psychiatric outcomes after the initiation of HC. Overall, our meta-analysis demonstrated a small yet statistically significant increase in mood-related morbidity (principally depressive symptoms and suicidality) following HC use, whereas associations with anxiety and other psychiatric disorders were less consistent. Quality stratification revealed that when analyses were restricted to high-quality cohorts, effect sizes were attenuated but remained positive, suggesting a signal that is modest in magnitude but robust in methodological rigor.

### 4.1 Comparison with prior work

The present findings of an increased risk of depressive outcomes following hormonal contraception initiation align with emerging evidence suggesting multiple interconnected neurobiological mechanisms underlying this association. The disruption of allopregnanolone synthesis represents a particularly compelling pathway, as preclinical studies demonstrate that ethinylestradiol and levonorgestrel combinations significantly reduce brain allopregnanolone concentrations by lowering the levels of its precursor hormone, progesterone, subsequently altering GABAA receptor subunit expression and increasing anxiety-like behaviors (59, 60). This neurosteroid, which serves as a positive allosteric modulator of GABA-A receptors, plays an important role in mood regulation, and its rapid decline has been implicated in depressive symptomatology through impaired GABAergic neurotransmission (61). Complementing this neurosteroid hypothesis, oral contraceptives induce stress-like alterations in the HPA axis, characterized by elevated basal cortisol levels and blunted stress reactivity, potentially due to ethinylestradiol increasing corticosteroid binding protein and reducing free cortisol availability (62). Notably, dysregulation of the HPA axis has been implicated in the development and persistence of psychopathological symptoms (63). Given these findings, future research should more closely examine hormonal contraceptive-induced dysregulation of the HPA axis, as such alterations have been associated with the emergence and persistence of psychiatric symptom. Our findings reveal stronger associations with both progestin-only methods and adolescent populations. The heightened susceptibility observed in adolescents may reflect developmental vulnerabilities in neurobiological systems involved in affect regulation, while the greater impact of progestin-only formulations may be attributable to their specific pharmacodynamic profiles (15). The observed heterogeneity in effect sizes across studies likely reflects the complex interplay between individual genetic predisposition, contraceptive formulation, and baseline psychiatric vulnerability, underscoring the need for personalized approaches to contraceptive counseling that incorporate mental health screening and monitoring protocols.

The present analysis revealed inconsistent evidence for associations between hormonal contraception and anxiety outcomes, with our findings that highlighted a non-significant pooled risk ratio of 1.08 [0.83-1.40], which aligns with recent systematic reviews suggesting mixed findings for anxiogenic effects of hormonal contraceptives. This variability may reflect the complex neurobiological mechanisms underlying anxiety regulation, where hormonal contraceptives simultaneously disrupt multiple neurotransmitter systems with potentially opposing effects on anxiety phenotypes. While the suppression of endogenous progesterone and its anxiolytic metabolite allopregnanolone could theoretically increase anxiety through reduced GABAergic tone, hormonal contraceptives, particularly OCs, may concurrently influence serotonergic pathways and other neurosteroid-mediated mechanisms that could have protective effects against anxiety in certain populations. The inconsistent findings across studies, particularly the divergent results showing increased panic disorder risk among LNG-IUS users (58) and cross-sectional analyses suggesting lower anxiety scores in combined oral contraceptive users (26), likely reflect the heterogeneous nature of anxiety disorders themselves, which encompass distinct phenotypes (generalized anxiety, panic disorder, social anxiety) that may respond differently to hormonal perturbations. Additionally, the predominant reliance on self-reported screening instruments rather than clinician-verified diagnoses, combined with inadequate control for psychiatric comorbidities and baseline anxiety vulnerability, may have obscured genuine associations. These methodological limitations, coupled with the potential for gene-environment interactions specific to anxiety phenotypes that require particular hormonal contexts for expression, underscore the need for well-powered prospective studies employing structured diagnostic interviews and comprehensive assessment of anxiety subtypes to definitively characterize the relationship between hormonal contraception and anxiety disorders.

**Figure 3:**
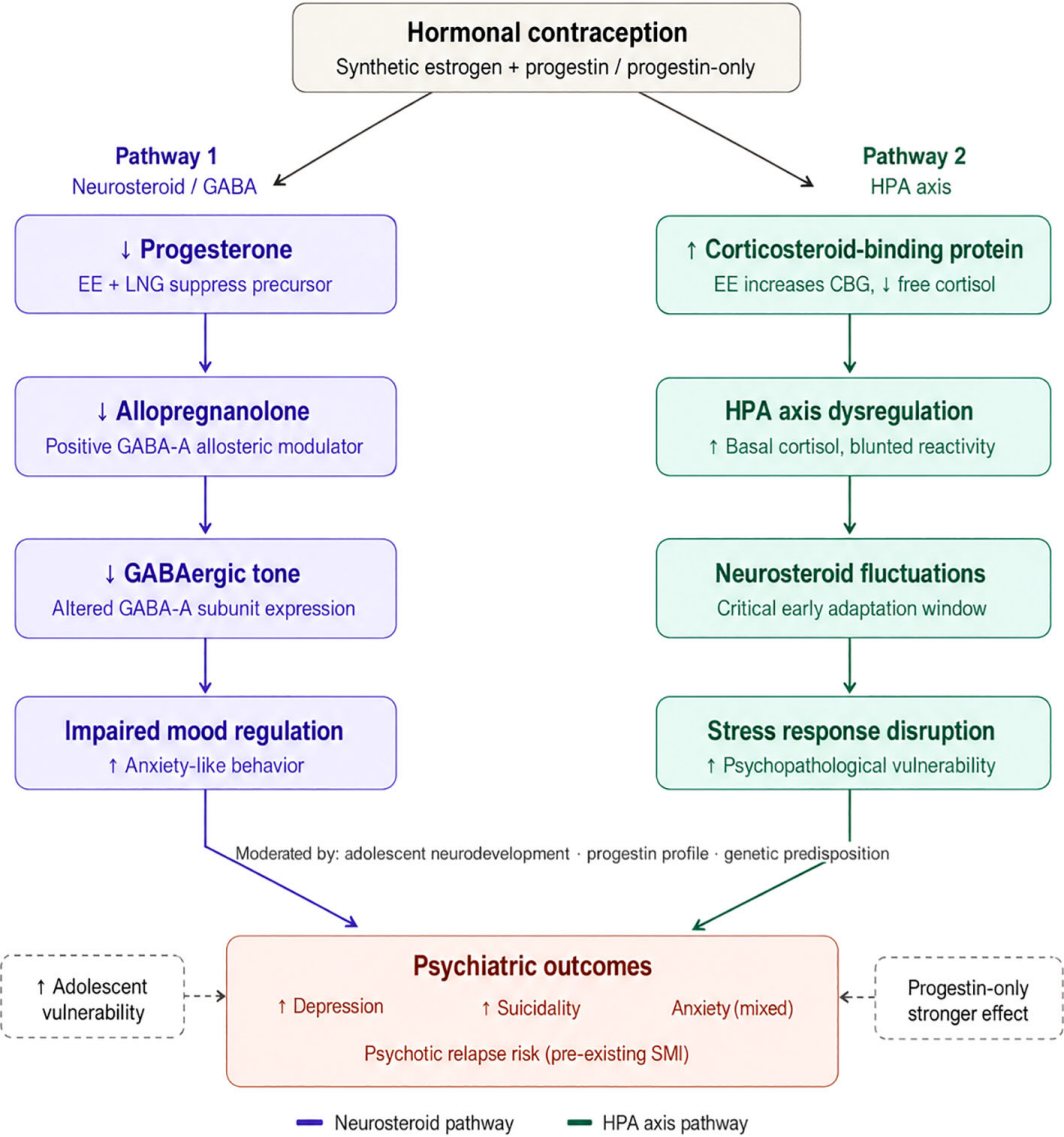
Proposed biological mechanisms linking hormonal contraception to psychiatric outcomes.

The evidence for associations between hormonal contraception and severe psychiatric disorders other than depression reveals a complex and nuanced landscape. Our findings regarding suicidality, while showing a non-significant pooled risk ratio of 1.20 [0.65-2.21], align with concerns raised by high-quality Danish registry data demonstrating elevated suicide attempt rates, particularly among adolescents within the first two months of hormonal contraception initiation (12). This temporal pattern suggests that the early adaptation period may represent a critical window of vulnerability, potentially mediated by rapid neurosteroid fluctuations and HPA-axis dysregulation that disproportionately affect adolescent neurobiological systems still undergoing maturation. The lack of evidence for increased bipolar disorder conversions among hormonal contraceptive users, as demonstrated by Finnish national database analyses (64), contrasts with the modest increase in psychotic relapses observed during DMPA therapy in women with established schizophrenia (8), suggesting that pre-existing severe mental illness may confer heightened susceptibility to hormonal perturbations. These differential effects underscore the importance of contraceptive selection based on individual psychiatric risk profiles and baseline symptomatology. Possible biological mechanisms are presented in **Figure 3**.

### 4.2 Recommendations and implications

The evidence synthesized in this review indicates a modest but clinically relevant elevation in depressive and, to a lesser extent, suicidality outcomes after the initiation of hormonal contraception (particularly progestin-only formulations in younger users) while pooled estimates for anxiety and other psychiatric disorders remain inconclusive. Accordingly, we recommend that prescribers adopt a stepped, mental-health-sensitive counseling model. First, a brief standardized screen (e.g., PHQ-9 and GAD-7) should be completed before starting any hormonal method and repeated at the 3- and 6-month follow-up visits, with results documented alongside adverse-event reporting. Second, where possible, women with a personal or first-degree family history of mood disorder should be offered non-hormonal or low-androgenic combined options (e.g., drospirenone/ethinylestradiol) as first-line contraception, reserving progestin-only methods for cases with clear medical indications. Third, collaborations between primary-care, gynecology, and mental-health services should be formalized to facilitate rapid treatment modification (dose reduction, switch to copper IUD, or short-term psychotropic therapy) if new-onset or worsening symptoms emerge. From a public-health perspective, pharmacovigilance systems should incorporate routine psychiatric signal detection for all newly marketed contraceptives, and regulatory agencies ought to require post-marketing mood-outcome surveillance analogous to that mandated for thromboembolic events. Finally, future research should prioritize prospective, formulation-specific trials that stratify by developmental stage and baseline vulnerability, and report standardized, clinician-verified psychiatric endpoints, to sharpen risk-benefit calculations for both prescribers and users.

### 4.3 Limitations

Several limitations should be taken into considerations. First, despite rigorous screening, the evidence base is dominated by observational designs, leaving residual confounding (particularly by indication and prior psychiatric history) largely unresolved. Second, heterogeneity was substantial (I² > 95%), driven in part by one pharmacovigilance outlier and several low-quality cross-sectional surveys; although sensitivity analyses attenuated the pooled estimate, between-study variance remained high. Third, psychiatric outcomes were often self-reported or inferred from medication proxies, raising concerns about misclassification. Fourth, our exclusion of studies focusing on polycystic ovary syndrome, endometriosis or HIV (conditions frequently managed with HC) may limit generalizability to those populations. Finally, language restrictions to English and French and a 10-year search window may have omitted earlier or non-Western literature. These limitations highlight the need for well-controlled, diagnosis-confirmed prospective cohorts and randomized trials that compare specific HC formulations while accounting for baseline mental-health risk.

## 5 Conclusion

This scoping review synthesizing data from 46 studies highlights a consistent, but modest, association between HC use (particularly progestin-only formulations) and increased risk of depressive symptoms and suicidality, especially among adolescent and early adult users. While the evidence for anxiety and other psychiatric outcomes remains inconclusive due to methodological heterogeneity and inconsistent endpoint definitions, the cumulative findings emphasize the importance of adopting a biopsychosocial lens when prescribing HCs. Notably, the quality assessment revealed considerable variation in study design rigor, with confounding control and psychiatric outcome specification emerging as recurrent limitations. These results suggest that psychiatric risk stratification and post-initiation monitoring should become integral components of contraceptive counseling, especially in populations with pre-existing or familial mental health vulnerabilities. Moving forward, there is a pressing need for high-quality, prospective, formulation-specific research that integrates validated psychiatric measures, considers neuroendocrine and developmental moderators, and includes both clinical and subclinical mental health endpoints. Interdisciplinary research that bridges reproductive endocrinology, psychiatry, and epidemiology (while leveraging innovations in digital health tracking and real-world data) may be particularly well-positioned to disentangle causal pathways and optimize contraceptive safety. A more personalized, evidence-informed approach to hormonal contraception will be important in advancing both reproductive autonomy and mental health equity across diverse populations.
