# Supplementary material for: Identifying psychiatric comorbidities that occur following the introduction of hormonal contraception: a scoping review
Source: Front Psychiatry. 2026 Jun 1;17:1783906. doi: 10.3389/fpsyt.2026.1783906 (PMC13265461; doi:10.3389/fpsyt.2026.1783906)
Supplement: Supplementary file 1 [file Table1.docx]

Supplementary Material

# Supplementary Tables

## Supplementary Table 1. PRISMA-ScR Checklist

| **SECTION** | **ITEM** | **PRISMA-ScR CHECKLIST ITEM** | **REPORTED ON PAGE #** |
| --- | --- | --- | --- |
| **TITLE** | | | |
| Title | 1 | Identify the report as a scoping review. | Title page |
| **ABSTRACT** | | | |
| Structured summary | 2 | Provide a structured summary that includes (as applicable): background, objectives, eligibility criteria, sources of evidence, charting methods, results, and conclusions that relate to the review questions and objectives. | Title page |
| **INTRODUCTION** | | | |
| Rationale | 3 | Describe the rationale for the review in the context of what is already known. Explain why the review questions/objectives lend themselves to a scoping review approach. | Introduction |
| Objectives | 4 | Provide an explicit statement of the questions and objectives being addressed with reference to their key elements (e.g., population or participants, concepts, and context) or other relevant key elements used to conceptualize the review questions and/or objectives. | Introduction |
| **METHODS** | | | |
| Protocol and registration | 5 | Indicate whether a review protocol exists; state if and where it can be accessed (e.g., a Web address); and if available, provide registration information, including the registration number. | N/A |
| Eligibility criteria | 6 | Specify characteristics of the sources of evidence used as eligibility criteria (e.g., years considered, language, and publication status), and provide a rationale. | Methods |
| Information sources* | 7 | Describe all information sources in the search (e.g., databases with dates of coverage and contact with authors to identify additional sources), as well as the date the most recent search was executed. | Methods |
| Search | 8 | Present the full electronic search strategy for at least 1 database, including any limits used, such that it could be repeated. | Methods |
| Selection of sources of evidence† | 9 | State the process for selecting sources of evidence (i.e., screening and eligibility) included in the scoping review. | Methods |
| Data charting process‡ | 10 | Describe the methods of charting data from the included sources of evidence (e.g., calibrated forms or forms that have been tested by the team before their use, and whether data charting was done independently or in duplicate) and any processes for obtaining and confirming data from investigators. | Methods |
| Data items | 11 | List and define all variables for which data were sought and any assumptions and simplifications made. | Methods |
| Critical appraisal of individual sources of evidence§ | 12 | If done, provide a rationale for conducting a critical appraisal of included sources of evidence; describe the methods used and how this information was used in any data synthesis (if appropriate). | Methods |
| Synthesis of results | 13 | Describe the methods of handling and summarizing the data that were charted. | Methods |
| **RESULTS** | | | |
| Selection of sources of evidence | 14 | Give numbers of sources of evidence screened, assessed for eligibility, and included in the review, with reasons for exclusions at each stage, ideally using a flow diagram. | Results |
| Characteristics of sources of evidence | 15 | For each source of evidence, present characteristics for which data were charted and provide the citations. | Results |
| Critical appraisal within sources of evidence | 16 | If done, present data on critical appraisal of included sources of evidence (see item 12). | Results |
| Results of individual sources of evidence | 17 | For each included source of evidence, present the relevant data that were charted that relate to the review questions and objectives. | Results |
| Synthesis of results | 18 | Summarize and/or present the charting results as they relate to the review questions and objectives. | Results |
| **DISCUSSION** | | | |
| Summary of evidence | 19 | Summarize the main results (including an overview of concepts, themes, and types of evidence available), link to the review questions and objectives, and consider the relevance to key groups. | Discussion |
| Limitations | 20 | Discuss the limitations of the scoping review process. | Discussion |
| Conclusions | 21 | Provide a general interpretation of the results with respect to the review questions and objectives, as well as potential implications and/or next steps. | Conclusions |
| **FUNDING** | | | |
| Funding | 22 | Describe sources of funding for the included sources of evidence, as well as sources of funding for the scoping review. Describe the role of the funders of the scoping review. | Funding statement |

JBI = Joanna Briggs Institute; PRISMA-ScR = Preferred Reporting Items for Systematic reviews and Meta-Analyses extension for Scoping Reviews.

* Where *sources of evidence* (see second footnote) are compiled from, such as bibliographic databases, social media platforms, and Web sites.

† A more inclusive/heterogeneous term used to account for the different types of evidence or data sources (e.g., quantitative and/or qualitative research, expert opinion, and policy documents) that may be eligible in a scoping review as opposed to only studies. This is not to be confused with *information sources* (see first footnote).

‡ The frameworks by Arksey and O’Malley (6) and Levac and colleagues (7) and the JBI guidance (4, 5) refer to the process of data extraction in a scoping review as data charting*.*

§ The process of systematically examining research evidence to assess its validity, results, and relevance before using it to inform a decision. This term is used for items 12 and 19 instead of "risk of bias" (which is more applicable to systematic reviews of interventions) to include and acknowledge the various sources of evidence that may be used in a scoping review (e.g., quantitative and/or qualitative research, expert opinion, and policy document).

*From:* Tricco AC, Lillie E, Zarin W, O'Brien KK, Colquhoun H, Levac D, et al. PRISMA Extension for Scoping Reviews (PRISMAScR): Checklist and Explanation. Ann Intern Med. 2018;169:467–473. [doi: 10.7326/M18-0850](http://annals.org/aim/fullarticle/2700389/prisma-extension-scoping-reviews-prisma-scr-checklist-explanation)

## Search strings:

**PUBMED**

("Contraceptive Agents, Hormonal"[Mesh] OR "Contraceptives, Oral"[Mesh] OR "Hormonal Contraception"[Mesh] OR "Long-Acting Reversible Contraception"[Mesh] OR ("Contraception"[Mesh:NoExp] AND ("Hormones"[Mesh:NoExp] OR "Progestins"[Mesh] OR "Estrogens"[Mesh])) OR "contraception hormonal"[TIAB:~4] OR "contraception hormone"[TIAB:~4] OR "contraception hormones"[TIAB:~4] OR "contraception pill"[TIAB:~4] OR "contraception pills"[TIAB:~4] OR "contraception oral"[TIAB:~4] OR "contraception injectable"[TIAB:~4] OR "contraception injectables"[TIAB:~4] OR "contraception implant"[TIAB:~4] OR "contraception implants"[TIAB:~4] OR "contraception combined"[TIAB:~4] OR "contraception progestin"[TIAB:~4] OR "contraception progestins"[TIAB:~4] OR "contraception estrogen"[TIAB:~4] OR "contraception estrogens"[TIAB:~4] OR "contraception oestrogen"[TIAB:~4] OR "contraception oestrogens"[TIAB:~4] OR "contraception progestogen"[TIAB:~4] OR "contraception progestogens"[TIAB:~4] OR "contraceptive hormonal"[TIAB:~4] OR "contraceptive hormone"[TIAB:~4] OR "contraceptive hormones"[TIAB:~4] OR "contraceptive pill"[TIAB:~4] OR "contraceptive pills"[TIAB:~4] OR "contraceptive oral"[TIAB:~4] OR "contraceptive injectable"[TIAB:~4] OR "contraceptive injectables"[TIAB:~4] OR "contraceptive implant"[TIAB:~4] OR "contraceptive implants"[TIAB:~4] OR "contraceptive combined"[TIAB:~4] OR "contraceptive progestin"[TIAB:~4] OR "contraceptive progestins"[TIAB:~4] OR "contraceptive estrogen"[TIAB:~4] OR "contraceptive estrogens"[TIAB:~4] OR "contraceptive oestrogen"[TIAB:~4] OR "contraceptive oestrogens"[TIAB:~4] OR "contraceptive progestogen"[TIAB:~4] OR "contraceptive progestogens"[TIAB:~4] OR "contraceptives hormonal"[TIAB:~4] OR "contraceptives hormone"[TIAB:~4] OR "contraceptives hormones"[TIAB:~4] OR "contraceptives pill"[TIAB:~4] OR "contraceptives pills"[TIAB:~4] OR "contraceptives oral"[TIAB:~4] OR "contraceptives injectable"[TIAB:~4] OR "contraceptives injectables"[TIAB:~4] OR "contraceptives implant"[TIAB:~4] OR "contraceptives implants"[TIAB:~4] OR "contraceptives combined"[TIAB:~4] OR "contraceptives progestin"[TIAB:~4] OR "contraceptives progestins"[TIAB:~4] OR "contraceptives estrogen"[TIAB:~4] OR "contraceptives estrogens"[TIAB:~4] OR "contraceptives oestrogen"[TIAB:~4] OR "contraceptives oestrogens"[TIAB:~4] OR "contraceptives progestogen"[TIAB:~4] OR "contraceptives progestogens"[TIAB:~4] OR "birth control hormonal"[TIAB:~4] OR "birth control hormone"[TIAB:~4] OR "birth control hormones"[TIAB:~4] OR "birth control pill"[TIAB:~4] OR "birth control pills"[TIAB:~4] OR "birth control oral"[TIAB:~4] OR "birth control injectable"[TIAB:~4] OR "birth control injectables"[TIAB:~4] OR "birth control implant"[TIAB:~4] OR "birth control implants"[TIAB:~4] OR "birth control combined"[TIAB:~4] OR "birth control progestin"[TIAB:~4] OR "birth control progestins"[TIAB:~4] OR "birth control estrogen"[TIAB:~4] OR "birth control estrogens"[TIAB:~4] OR "birth control oestrogen"[TIAB:~4] OR "birth control oestrogens"[TIAB:~4] OR "birth control progestogen"[TIAB:~4] OR "birth control progestogens"[TIAB:~4]) **AND** ("Psychiatry"[Mesh] OR "Mental Disorders"[Mesh:NoExp] OR "Mental Health"[Mesh] OR "Mood Disorders"[Mesh:NoExp] OR "Bipolar and Related Disorders"[Mesh] OR "Depressive Disorder"[Mesh] OR "Anxiety Disorders"[Mesh] OR "Schizophrenia Spectrum and Other Psychotic Disorders"[Mesh] OR "Personality Disorders"[Mesh] OR "Suicide"[Mesh] OR "Affective Symptoms"[Mesh] OR Psychiatry[TIAB] OR Psychiatric[TIAB] OR "Mental disorder*"[TIAB] OR "Mental illness*"[TIAB] OR "Mental health"[TIAB] OR Depression[TIAB] OR Depressive[TIAB] OR "mood disorder*"[TIAB] OR "Mood symptom"[TIAB:~3] OR "mood symptoms"[TIAB:~3] OR "Affective disorder*"[TIAB] OR "Affective symptom"[TIAB:~3] OR "affective symptoms"[TIAB:~3] OR "psychological disorder*"[TIAB] OR "Psychological symptom"[TIAB:~3] OR "psychological symptoms"[TIAB:~3] OR "emotional disorder*"[TIAB] OR "Emotional symptom"[TIAB:~3] OR "emotional symptoms"[TIAB:~3] OR "Bipolar disorder"[TIAB:~2] OR "bipolar disorders"[TIAB:~2] OR Anxiety[TIAB] OR Panic[TIAB] OR Phobic[TIAB] OR Phobia*[TIAB] OR "Obsessive compulsive"[TIAB] OR Psychosis[TIAB] OR Psychotic[TIAB] OR Schizo*[TIAB] OR "Personality disorder*"[TIAB] OR Suicide*[TIAB] OR Suicidal[TIAB])

**MEDLINE**

1. contraceptive agents, hormonal/ or contraceptives, oral/ or contraceptives, oral, combined/ or contraceptives, oral, hormonal/ or contraceptives, oral, sequential/ or contraceptives, oral, synthetic/

2. hormonal contraception/ or long-acting reversible contraception/

3. Contraception/

4. Hormones/

5. Progestins/

6. exp Estrogens/

7. or/4-6

8. and/3,7

9. ((contracept* or "birth control") adj4 (Hormon* or pill or pills or oral or injectable* or implant or implants or combined or progestin* or ?estrogen* or progestogen*)).ti,ab,kw.

10. or/1-2,8-9

11. exp psychiatry/ or exp community psychiatry/ or exp forensic psychiatry/

12. Mental Disorders/

13. Mental Health/

14. mood disorders/ or exp "bipolar and related disorders"/ or exp depressive disorder/

15. exp anxiety disorders/ or exp obsessive-compulsive disorder/ or exp phobic disorders/

16. exp "schizophrenia spectrum and other psychotic disorders"/ or exp psychotic disorders/ or exp schizophrenia/

17. exp Personality Disorders/

18. exp Suicide/

19. Affective Symptoms/

20. (Psychiatry or Psychiatric or "Mental disorder*" or "Mental illness*" or "Mental health" or Depression or Depressive or "mood disorder*" or (Mood adj3 symptom*) or "Affective disorder*" or (Affective adj3 symptom*) or "psychological disorder*" or (Psychological adj3 symptom*) or "emotional disorder*" or (Emotional adj3 symptom*) or (Bipolar adj2 disorder*) or Anxiety or Panic or Phobic or Phobia* or "Obsessive compulsive" or Psychosis or Psychotic or Schizo* or "Personality disorder*" or Suicide* or Suicidal).ti,ab,kw.

21. or/11-20

22. and/10,21

**EMBASE**

1. *hormonal contraceptive agent/ or *injectable contraceptive agent/ or *oral contraceptive agent/

2. *long-acting reversible contraception/

3. *hormonal contraception/

4. *oral contraception/

5. *contraception/

6. *hormone/

7. *gestagen/

8. *estrogen/

9. or/6-8

10. and/5,9

11. ((contracept* or "birth control") adj4 (Hormon* or pill or pills or oral or injectable* or implant or implants or combined or progestin* or ?estrogen* or progestogen*)).ti,ab,kw.

12. or/1-4,10-11

13. exp psychiatry/

14. mental disease/

15. exp mental health/ or community mental health/

16. mood disorder/ or chronic depression/ or depressive psychosis/ or major depression/ or major affective disorder/ or schizoaffective psychosis/

17. exp bipolar disorder/

18. anxiety disorder/ or generalized anxiety disorder/ or exp obsessive compulsive disorder/ or panic/ or exp phobia/

19. psychosis/ or exp schizophrenia/

20. exp schizophrenia spectrum disorder/

21. exp personality disorder/ or exp cluster a personality disorder/ or exp cluster b personality disorder/ or exp cluster c personality disorder/

22. suicidal behavior/ or suicidal ideation/ or suicide/ or suicide attempt/

23. exp emotional disorder/

24. (Psychiatry or Psychiatric or "Mental disorder*" or "Mental illness*" or "Mental health" or Depression or Depressive or "mood disorder*" or (Mood adj3 symptom*) or "Affective disorder*" or (Affective adj3 symptom*) or "psychological disorder*" or (Psychological adj3 symptom*) or "emotional disorder*" or (Emotional adj3 symptom*) or (Bipolar adj2 disorder*) or Anxiety or Panic or Phobic or Phobia* or "Obsessive compulsive" or Psychosis or Psychotic or Schizo* or "Personality disorder*" or Suicide* or Suicidal).ti,ab,kw.

25. or/13-24

26. and/12,25

27. limit 26 to (article or article in press or "preprint (unpublished, non-peer reviewed)" or "review")

**EBM Reviews – Cocrane Database of Systematic Reviews**

1. ((contracept* or "birth control") adj4 (Hormon* or pill or pills or oral or injectable* or implant or implants or combined or progestin* or ?estrogen* or progestogen*)).ti,ab.

2. (Psychiatry or Psychiatric or "Mental disorder*" or "Mental illness*" or "Mental health" or Depression or Depressive or "mood disorder*" or (Mood adj3 symptom*) or "Affective disorder*" or (Affective adj3 symptom*) or "psychological disorder*" or (Psychological adj3 symptom*) or "emotional disorder*" or (Emotional adj3 symptom*) or (Bipolar adj2 disorder*) or Anxiety or Panic or Phobic or Phobia* or "Obsessive compulsive" or Psychosis or Psychotic or Schizo* or "Personality disorder*" or Suicide* or Suicidal).ti,ab.

3. and/1-2

**PsycINFO**

1. oral contraceptives/

2. birth control/

3. hormones/

4. exp progestational hormones/

5. exp estrogens/

6. or/3-5

7. and/2,6

8. ((contracept* or "birth control") adj4 (Hormon* or pill or pills or oral or injectable* or implant or implants or combined or progestin* or ?estrogen* or progestogen*)).ti,ab,id.

9. or/1,7-8

10. exp psychiatry/

11. mental disorders/

12. mental health/

13. serious mental illness/

14. exp affective disorders/ or exp major depression/

15. exp bipolar disorder/

16. anxiety disorders/ or generalized anxiety disorder/ or panic attack/ or panic disorder/ or exp phobias/

17. exp obsessive compulsive disorder/

18. psychosis/ or affective psychosis/ or chronic psychosis/ or delusional disorder/ or exp paranoid psychosis/ or exp schizophrenia/

19. exp personality disorders/

20. exp suicidal behavior/ or exp suicide/ or suicidality/

21. externalizing symptoms/ or internalizing symptoms/ or psychiatric symptoms/

22. (Psychiatry or Psychiatric or "Mental disorder*" or "Mental illness*" or "Mental health" or Depression or Depressive or "mood disorder*" or (Mood adj3 symptom*) or "Affective disorder*" or (Affective adj3 symptom*) or "psychological disorder*" or (Psychological adj3 symptom*) or "emotional disorder*" or (Emotional adj3 symptom*) or (Bipolar adj2 disorder*) or Anxiety or Panic or Phobic or Phobia* or "Obsessive compulsive" or Psychosis or Psychotic or Schizo* or "Personality disorder*" or Suicide* or Suicidal).ti,ab,id.

23. or/10-22

24. and/9,23

25. limit 24 to ("0100 journal" or "0110 peer-reviewed journal" or "0120 non-peer-reviewed journal" or "0130 peer-reviewed status unknown")
