# Supplementary material for: Identifying psychiatric comorbidities that occur following the introduction of hormonal contraception: a scoping review
Source: Front Psychiatry. 2026 Jun 1;17:1783906. doi: 10.3389/fpsyt.2026.1783906 (PMC13265461; doi:10.3389/fpsyt.2026.1783906)
Supplement: Supplementary file 2 [file Table2.docx]

# **JBI Critical Appraisal Table**

## Legend

- Y = Yes
- N = No
- U = Unclear
- NA = Not applicable

Table S2. Cohort Studies - JBI Critical Appraisal Checklist

| Study | Groups Similar/Recruited Same Population | Exposure Measured Validly | Exposure Measured Reliably | Confounders Identified | Strategies for Confounders | Participants Free of Outcome at Baseline | Outcomes Measured Validly/Reliably | Follow-up Sufficient | Follow-up Complete | Incomplete Follow-up Addressed | Appropriate Statistical Analysis | Overall Appraisal |
| --- | --- | --- | --- | --- | --- | --- | --- | --- | --- | --- | --- | --- |
| Skovlund et al., 2018 | Y | Y | Y | Y | Y | Y | Y | Y | Y | NA | Y | High |
| Zettermark et al., 2018 | Y | Y | Y | Y | Y | Y | Y | Y | Y | NA | Y | High |
| de Wit et al., 2020 | Y | Y | Y | U | U | Y | Y | Y | U | U | Y | Moderate |
| Anderl et al., 2022 | Y | Y | Y | Y | Y | Y | Y | Y | U | U | Y | High |
| Edwards et al., 2022 | Y | Y | Y | Y | Y | Y | Y | Y | Y | NA | Y | High |
| Drake et al., 2020 | Y | Y | Y | Y | U | Y | Y | Y | U | U | Y | Moderate |
| Larsen et al., 2023 | Y | Y | Y | Y | Y | Y | Y | Y | Y | NA | Y | High |
| Skovlund et al., 2024 | Y | Y | Y | Y | Y | Y | Y | Y | Y | NA | Y | High |
| Doornweerd et al., 2022 | Y | Y | Y | U | U | Y | Y | Y | U | U | Y | Moderate |
| Ross et al., 2021 | Y | Y | Y | Y | U | Y | Y | Y | U | U | Y | Moderate |
| Roberts and Hansen, 2017 | Y | Y | Y | Y | Y | Y | Y | Y | Y | NA | Y | High |
| Morssinkhof et al., 2021 | Y | Y | Y | Y | Y | N | Y | Y | U | U | Y | Moderate |
| Zettermark et al., 2021 | Y | Y | Y | Y | Y | U | Y | Y | Y | NA | Y | High |

# Cross-Sectional Studies - JBI Critical Appraisal Checklist

| Study | Inclusion Criteria Clearly Defined | Subjects/Setting Described | Exposure Measured Validly/Reliably | Standard Criteria Used for Outcome | Confounders Identified | Strategies for Confounders | Outcomes Measured Validly/Reliably | Appropriate Statistical Analysis | Overall Appraisal |
| --- | --- | --- | --- | --- | --- | --- | --- | --- | --- |
| Gregory et al., 2018 | Y | Y | U | U | U | U | U | Y | Moderate |
| Stewart et al., 2022 | Y | Y | Y | Y | U | U | Y | Y | Moderate |
| Lawley et al., 2018 | Y | Y | Y | Y | Y | Y | Y | Y | High |
| Yusuf et al., 2024 | Y | Y | Y | Y | Y | Y | Y | Y | High |
| Masama et al., 2022 | Y | Y | Y | Y | U | U | Y | Y | Moderate |
| Cheslack-Postava et al., 2014 | Y | Y | Y | Y | Y | Y | Y | Y | High |
| Lewandowski et al., 2020 | Y | Y | Y | Y | U | U | Y | Y | Moderate |
| Thamkhantho et al., 2020 | U | Y | U | N | U | N | U | U | Low |
| Albawardi et al., 2022 | Y | Y | Y | Y | Y | Y | Y | Y | High |
| Kowalczyk et al., 2024 | Y | Y | Y | Y | Y | Y | Y | Y | High |
| Newman, 2022 | Y | Y | Y | Y | U | U | Y | Y | Moderate |
| Gawronska et al., 2024 (SI) | Y | Y | Y | Y | Y | Y | Y | Y | High |
| Roomaney and Lourens, 2020 | Y | Y | Y | Y | Y | U | Y | Y | Moderate |
| Smith et al., 2018 | Y | Y | Y | Y | Y | U | Y | Y | Moderate |
| Al-Deresawi and Habeeb, 2020 | U | Y | U | N | U | N | U | U | Low |
| Alfaifi et al., 2021 | Y | Y | Y | Y | U | U | Y | Y | Moderate |
| Déa et al., 2024 | Y | Y | Y | Y | Y | Y | Y | Y | High |
| Gawronska et al., 2024 (MDD) | Y | Y | Y | Y | Y | Y | Y | Y | High |
| Oxfeldt et al., 2020 | Y | Y | U | U | U | U | U | Y | Moderate |

# Randomized Controlled Trials - JBI Critical Appraisal Checklist

| Study | True Randomization | Allocation Concealed | Groups Similar at Baseline | Participants Blinded | Treatment Providers Blinded | Outcome Assessors Blinded | Groups Treated Identically | Follow-up Complete | Analyzed in Assigned Groups | Outcomes Measured Same Way | Outcomes Measured Reliably | Appropriate Statistical Analysis | Trial Design Appropriate | Overall Appraisal |
| --- | --- | --- | --- | --- | --- | --- | --- | --- | --- | --- | --- | --- | --- | --- |
| Shahnazi et al., 2014 | Y | U | Y | Y | U | U | Y | Y | U | Y | Y | Y | Y | Moderate |
| Lundin et al., 2017 | Y | Y | Y | Y | Y | Y | Y | Y | Y | Y | Y | Y | Y | High |
| Zethraeus et al., 2017 | Y | Y | Y | Y | Y | Y | Y | Y | Y | Y | Y | Y | Y | High |
| Bengtsdotter et al., 2018 | Y | Y | Y | Y | Y | Y | Y | Y | Y | Y | Y | Y | Y | High |

# Quasi-Experimental / Non-Randomized Comparative Studies - JBI Critical Appraisal Checklist

| Study | Clear Cause and Effect | Similar Participants | Similar Care Other Than Exposure | Control Group | Multiple Outcome Measurements | Complete Follow-up | Outcomes Measured Reliably | Appropriate Statistical Analysis | Overall Appraisal |
| --- | --- | --- | --- | --- | --- | --- | --- | --- | --- |
| Caruso et al., 2023 | Y | U | U | Y | Y | U | Y | Y | Moderate |
| Cetin et al., 2015 | Y | U | U | Y | Y | U | Y | Y | Moderate |
| Khafagy et al., 2021 | Y | Y | U | Y | Y | U | Y | Y | Moderate |
| Morotti et al., 2017 | Y | U | U | N | Y | U | Y | Y | Moderate |
| Ekenros et al., 2019 | Y | Y | Y | Y | Y | Y | Y | Y | High |
| Singata-Madliki et al., 2021 | Y | Y | Y | Y | Y | Y | Y | Y | High |
| Yonkers et al., 2017 | Y | Y | Y | Y | Y | Y | Y | Y | High |
